# Reduction in Accommodative Response of Schoolchildren by a Double-Mirror System

**DOI:** 10.3390/ijerph18199951

**Published:** 2021-09-22

**Authors:** Shang-Min Yeh, Chen-Cheng Lo, Chi-Hung Lee, Yu-Jung Chen, Feng-Chi Lin, Shuan-Yu Huang

**Affiliations:** 1 Department of Optometry, Chung Shan Medical University, Taichung 402, Taiwan; ysm@csmu.edu.tw (S.-M.Y.); s0885013@gm.csmu.edu.tw (C.-C.L.); 2 Department of Ophthalmology, Chung Shan Medical University Hospital, Taichung 402, Taiwan; 3 Department of Electrical Engineering, Feng Chia University, Taichung 407, Taiwan; chihlee@fcu.edu.tw; 4 Ph.D. Program of Electrical and Communications Engineering, Feng Chia University, Taichung 407, Taiwan; yujung@mail.dyu.edu.tw; 5 Department of Optometry, Da-Yeh University, Changhua 515, Taiwan; 6 Department of Ophthalmology, Kaohsiung Armed Forced General Hospital, Kaohsiung 802, Taiwan

**Keywords:** accommodative response, double-mirror system (DMS), accommodative relaxation, myopia control

## Abstract

Purpose: This study first proposed the application of a double-mirror system (DMS) to extend viewing distance and investigate the accommodative response of schoolchildren under a DMS. Method: Fifty-seven subjects aged between 7 and 12 years old were recruited in this study, and the experiment was divided into two stages. The first stage consisted of a case history inquiry, a refraction state, and a visual function examination. In the second stage, the subjects gazed at an object at distances of 0.4 m, 2.285 m, and through a DMS, respectively, and their accommodative responses were measured using an open-field autorefractor. Results: There was no significant difference in the schoolchildren’s accommodative response between subjects gazing at an object at 2.285 m (0.14 ± 0.35 D, *p* > 0.05) and those gazing at it through a DMS (0.20 ± 0.35 D). However, their accommodative response showed a significant difference between subjects gazing at an object at 0.4 m and 2.285 m and those gazing at it at 0.4 m and through a DMS. Conclusion: In this experiment, the results of the children’s accommodative response measured at 2.285 m or through a DMS are very similar. The viewing distance can be extended by a DMS, resulting in accommodative relaxation. This result may have potential applications in myopia control.

## 1. Introduction

Myopia is one of the most prevalent diseases in the world today. According to a study by Brien A. Holden and others, 2.620 billion people may have had myopia in 2020, with a myopia rate of 34.0% in the global population; by 2050, the global myopia population may be as high as 4.758 billion, representing a myopia rate of nearly half of the global population (49.8%) [1,2,3,4,5,6,7]. Myopia may lead to eye-related diseases, such as cataracts, glaucoma, retinal detachment, etc., and these diseases can cause irreversible visual impairment [3]. The rate of myopia has been increasing year by year, which makes it an important global issue that cannot be ignored. 

Although it is not clear exactly why some people are short-sighted, the cause of myopia may be the result of a combination of genetic or ethnic multi-factors. According to one study, cultural differences often result in extreme disparities in myopia rates, with eastern educational cultures allowing schoolchildren to spend less time outdoors and more time engaged in work, meaning activities conducted at a short working distance, such as reading, studying, etc. The time that children spend outdoors is lower in the developed countries of east and southeast Asia. Children of European origin spend over 21 h a week outdoors outside of school hours compared to the 14 h for children of east Asian origin aged 6–7, and the latter have a higher prevalence of myopia [5]. Many studies have shown that, among the factors that contribute to myopia, the duration of near-vision work and its intensity are most strongly associated with myopia. A study by Jenny M. Ip et al. noted that children who read at a distance of less than 30 cm away from a book, screen, etc. were more likely to be myopic than those who read at a distance of more than 30 cm away (*p* = 0.0003) [8,9,10,11,12,13,14,15]. The complex relationship between near-vision work and accommodation is mostly due to the inability to compensate for the defocusing of a retinal image, which can produce accommodation errors. Accommodation errors in near-vision work can produce hyperopic defocusing in the retina, which causes myopia and illustrates the importance of reading distance [16]. 

Collins described his open-view, infrared electronic refractionometer, and noted that, in some patients, “the signal indicating the focus is not stable, thus, accommodation is continually varying by a small amount at an unexpected speed; actually, a change equal to about 0.5 D plus or minus from the position of focus occurs about once per second or even faster” [17]. This finding has led many subsequent studies referring to such continuous accommodative variability as fluctuations in accommodation, or accommodative microfluctuations (AMFs) [17,18]. Harb et al. stated that unstable AMFs during continuous reading may produce blurred signals, leading to the onset of myopia [19].

Currently, computers, communication, and consumer electronics are popular, and the age of users is decreasing year by year. These electronic reading devices make viewing distances closer, which causes eye fatigue [20,21,22,23]. In order to reduce accommodation and slow the progress of myopia, this study proposes a new simple design of a double-mirror system (DMS) to extend viewing distance; while an object is still placed at a near distance, a virtual image is located further in front of the eye, resulting in accommodative relaxation.

## 2. Materials and Methods

### 2.1. Design of the Proposed a Double-Mirror System

This study first proposed a double-mirror system to extend viewing distance to result in accommodative relaxation. The system consists of two mirrors, one concave and one convex. The convex mirror first reduces the image and enlarges the field of view; then, the concave mirror enlarges the image. Finally, the image is viewed by the human eye. 

**Figure 1:**
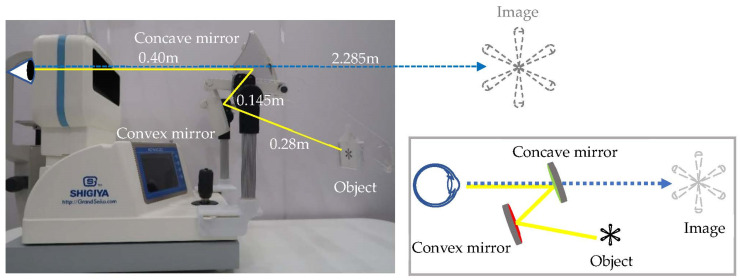
Experimental setup to measure the accommodative response using an open-field autorefractor as the dominant eye gazes at the image through a double-mirror system (DMS).

The diopters of the concave and convex mirrors are +2.83 D and −2.83 D, respectively. The distance between the human eye and the concave mirror is 400 mm, that between the concave mirror and the convex mirror is 145 mm, and that between the convex mirror and the object (Asterisk 3 × 3 cm) is 280 mm. Based on the simulation, the distance from the eye to the image can be up to 2.285 m, and the magnification of the image is 3.386 times, as shown in Figure 1. The optical defect of the DMS is the magnification difference between the horizontal and the vertical directions; it needs to be improved for further application.

### 2.2. Subjects

This study recruited 57 subjects from Yi-Chang primary school in Hualien and Chung-Cheng elementary school in Chu-Pei, Hsin-Chu county, furthermore, the ages were between seven and twelve years. The inclusion criteria were: those who had no prior eye or systemic diseases, a spherical refraction ranging from +1.0 D to −0.5 D, an astigmatism greater than −0.75 D, monocular and binocular distance visual acuity of 0.1 logMAR or better, and those with normal binocular vision.

Informed consent was obtained from all the subjects, and the experiment was conducted in accordance with the Declaration of Helsinki. Ethical approval was obtained from the Institutional Review Board of the Chung Shan Medical University Hospital (Taichung, Taiwan, ROC) (Approval number: CS1-20046).

### 2.3. Research Process

The experimental procedure included two steps. The first step was an examination of the basic visual function, where each subject received an initial examination of their refractive status, visual acuity, phoria, and stereoscopic vision. 

The second step was to measure the subjects’ dynamic accommodative responses and microfluctuations. The viewing distances were set for the experiment as follows: (1) the subjects gazed at a real object that was placed at distances of 0.4 m and 2.285 m; (2) they gazed at a virtual image that was located at a distance of 2.285 m through a double-mirror system (DMS). An open-field autorefractor (Grand Seiko WAM-5500) was used to measure the dynamic accommodative responses and microfluctuations of the subjects, and each detection time was 20 s. Only the data of the dominant eye were measured in the experiment, and the subjects were required to cover their nondominant eye with an occluder covering. During detection, the subjects were allowed to blink naturally, but they were asked to maintain their gaze at the object or image. The subjects were subjected to a two-stage refraction examination using an open-field autorefractor. First, the subjects’ refractive error at 6 m was measured. Second, the subjects’ refractive status at the given viewing distance (0.4 m, 2.285 m, or through a DMS) was measured. Accommodative response can be defined as the difference between the refractive statuses of the two-stage measurements, and can be represented as the following formula,

AR = RE − RS(1)

where AR is accommodative response, RE is the refractive error at 6 m, RS is the refractive status at the given viewing distance (0.4 m, 2.285 m, or through a DMS), and the unit of accommodative response is diopter (D).

### 2.4. Data Analysis

During the experiment, an open-field autorefractor was used to record the subjects’ dynamic accommodative responses and microfluctuations every 0.2 s. The dynamic accommodative responses and the microfluctuations of the dominant eye were recorded on a computer, all data were analyzed by SPSS Statistics 22, and independent sample t-testing and one-way ANOVA were conducted for statistical analysis.

## 3. Results

Among the 57 subjects, the dominant eye of 38 subjects was the right eye, and the remaining 19 had the left eye as the dominant eye. Regarding the baseline of the subjects—inclusive of the mean—spherical refraction was 0.28 ± 0.41 D, the mean cylindrical refraction was −0.40 ± 0.24 D, the best visual acuity value was −0.06 ± 0.07 logMAR, and the average amplitude of accommodation was 17.75 ± 5.16 D, as shown in Table 1.

Table 2 shows the gender differences (28 males, 29 females) in accommodative response. The results of the independent sample t-test show that the accommodative responses for the male schoolchildren were slightly greater than those of the female schoolchildren at viewing distances of 0.4 m, 2.285 m, and through a DMS. However, there was no statistically significant difference in terms of gender.

The subjects were divided into three groups: Lower Grade (7–8 years old), Middle Grade (9–10 years old), and Higher Grade (11–12 years old). The subjects gazed at the object at the different distances of 0.4 m, 2.285 m, and through a DMS, and the accommodative responses were measured. The accommodative responses for these three groups were very similar when the subjects gazed at the same viewing distance or gazed at the image through a DMS.

According to the Ministry of Education of Taiwan, younger schoolchildren in grades one and two have fewer learning hours than those in grades five and six. The difference is seven hours within a week between lower and higher grades, which is the reason we divided them to three age groups. We assumed the different school learning times may affect accommodative response; therefore, we divided them for this experiment. The result of a one-way ANOVA showed that accommodative response was not significantly related to schoolchildren’s grade, as shown in Table 3. 

**Figure 2:**
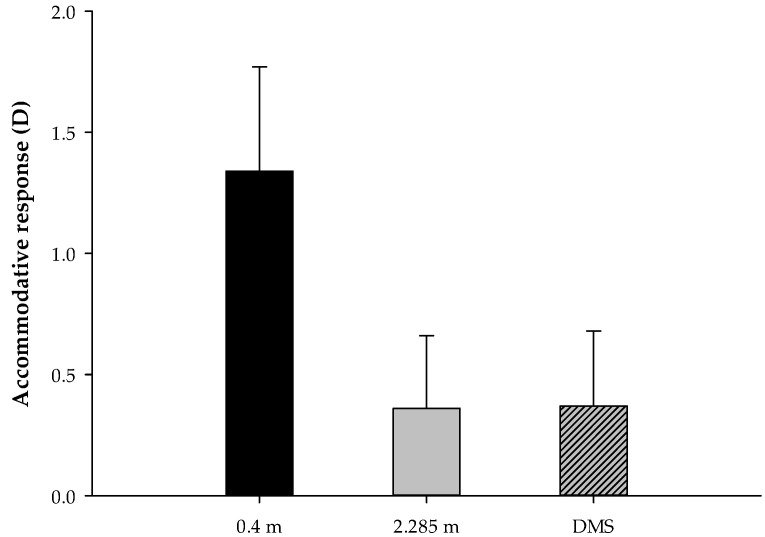
The accommodative responses of the different viewing distances of 0.4 m, 2.285 m, and through a DMS.

The average accommodative responses of all subjects were 1.34 ± 0.43 D, 0.36 ± 0.30 D, and 0.37 ± 0.31 D, which correspond to the subjects gazing at an object at 0.4 m, 2.285 m, and through a DMS, respectively. The one-way ANOVA results show that there was a significant difference in the different viewing distances (F(2, 168) = 186.942, *p* < 0.001). Post hoc tests with Bonferroni correction showed that the accommodative response at 0.4 m was significantly greater than that at 2.285 m (*p* < 0.001) or through a DMS (*p* < 0.001). However, there was no significant difference in the schoolchildren’s accommodative responses when they gazed at the object at 2.285 m or through a DMS (*p* > 0.05), as shown in Figure 2. 

**Figure 3:**
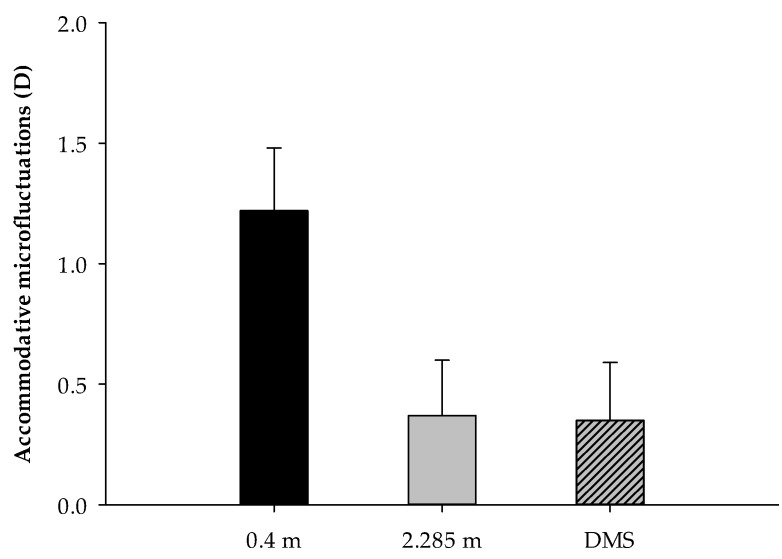
The accommodative microfluctuations (AMFs) at the viewing distances of 0.4 m, 2.285 m, and through a DMS.

The root-mean-square values of the accommodative microfluctuations (AMFs) were 1.22 ± 0.26 D, 0.37 ± 0.23 D, and 0.35 ± 0.24 D, which correspond to the subjects gazing at the object at 0.4 m, 2.285 m, and through a DMS, respectively [17]. The one-way ANOVA results show that there was significant difference in the viewing distances [F(2, 168) = 240.586, *p* < 0.001]. Post hoc tests with Bonferroni correction show that the accommodative microfluctuations of the schoolchildren at 0.4 m were significantly greater than at 2.285 m (*p* < 0.001) and through the double-mirror system (*p* < 0.001). However, there was no significant difference in the schoolchildren’s accommodative microfluctuations when they gazed at the object at 2.285 m or through a DMS (*p* > 0.05), as shown in Figure 3. 

**Figure 4:**
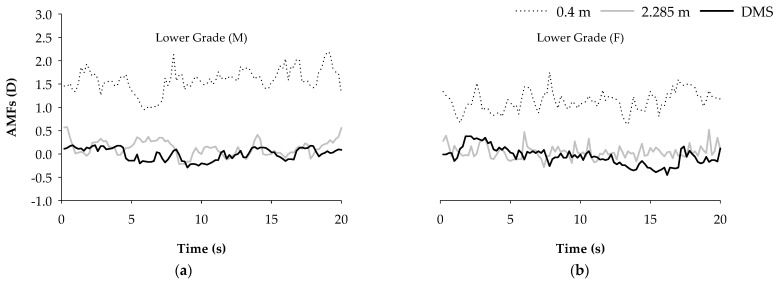

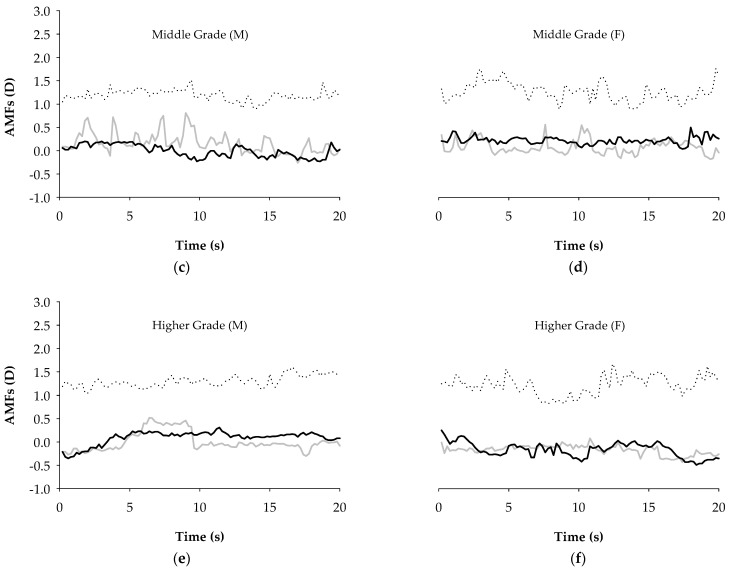
The changes in the accommodative microfluctuations for one male and one female schoolchild selected randomly from lower, middle and higher grades at different viewing distances of 0.4 m, 2.285 m, and through a DMS. (**a**,**c**,**e**) selected one boy from each group as lower, middle and higher grades. (**b**,**d**,**f**) selected one girl from each group, based on the above groups.

In order to further understand the changes in the accommodative microfluctuations at different viewing distances of 0.4 m, 2.285 m, and through a DMS, this study randomly selected one male and one female subject from each of the lower, middle, and higher grades to conduct an analysis of the accommodative response in 20 s. The results show that the accommodative microfluctuations were stable at the viewing distance of 2.285 m and through a DMS; on the contrary, the accommodative microfluctuation was unstable at the viewing distance of 0.4 m, as shown in Figure 4.

## 4. Discussion

There is much evidence suggesting that accommodation is a key factor in the development of myopia, although its exact mechanism is yet to be determined [24,25,26]. Retinal hyperopic defocus is regarded as a possible cause contributing to myopia, as is the link between near-vision work and myopia. Retinal hyperopic defocus can be induced by accommodative lag during near-vision work. Thus, accommodative lag has been proposed to promote axial elongation [27]. Intense near-vision work tasks may lead to episodes of transient myopia and the development of permanent myopia [28]. In this study, we developed a double-mirror system (DMS) to extend viewing distance, which can reduce the lag in accommodation and hyperopia defocus and, as a result, may help to slow the development of myopia. The lag in accommodation at 0.4 m, 2.285 m, and through a DMS was measured. The accommodative lag was considered to be derived from an imperfection in the neural integrator in the accommodation control system; however, this study considered that the subjects were rarely aware of image blur because the lag in accommodation did not usually surpass the focal depth of the eye (usually within ±0.5D) [29,30,31,32]. The values of the accommodative lag were 1.16D, 0.07D, and 0.06D, which correspond to the subjects gazing at the object at 0.4 m, 2.285 m, and through a DMS, respectively. As the accommodative lag was smaller at 2.285 m and through a DMS, it did not cause an obvious blur stimulus. The reason for the larger accommodative lag of 1.16D at 0.4 m was speculated to be due to the immature stability of the schoolchildren’s refractive state [33].

While the accommodative responses for the subjects gazing at the object both at a distance of 2.285 m and through a DMS were similar, there was no statistically significant difference, which means that a viewing distance of 2.285 m can be achieved through a DMS, resulting in accommodative relaxation. The accommodative microfluctuations in schoolchildren at 0.4 m were significantly greater than at 2.285 m and in the double-mirror system. As a greater accommodative response is needed at the near distance of 0.4 m, unstable AMFs may be generated. The accommodative microfluctuations for the schoolchildren gazing at the object at 2.285 m and through DMS were very close, at 0.37D and 0.35D, respectively. The stability of AMFs when gazing at an image through a DMS was consistent with the results when gazing at the same object at 2.285 m.

This study investigated accommodative response at different viewing distances and through a DMS. To avoid a convergence effect, which affects the accommodative response, we allowed the subjects to perform monocular fixation; therefore, there are some limitations in this study. The results show that accommodative relaxation and microfluctuation stability can be achieved by a double-mirror system.

## 5. Conclusions

This study measured the accommodative response and microfluctuations in schoolchildren at different viewing distances of 0.4 m, 2.285 m, and through a DMS. The accommodative responses were 1.34 ± 0.43 D, 0.36 ± 0.30 D, and 0.37 ± 0.31 D; the accommodative microfluctuations were 1.22 ± 0.26 D, 0.37 ± 0.23 D, and 0.35 ± 0.24 D, which correspond to viewing distances of 0.4 m, 2.285 m, and through a DMS. 

The values of the accommodative responses and microfluctuations for the near-viewing distance of 0.4 m were larger than 0.97 D and 0.87 D, as well as those through a DMS. 

This result shows that the accommodative response can indeed reduce and stabilize microfluctuations through a DMS. In addition, the values of the accommodative response and microfluctuations for both the viewing distance of 2.285 m and through a DMS are similar, and the viewing distance can be extended through a DMS; in addition, the magnification is set to 3.386 times. The simple optical design of a double-mirror system (DMS) have potential for reducing the lag in accommodation and hyperopia defocus, and could result in slowing the development of myopia. 

## Figures and Tables

**Table 1:** The baseline of the subjects.

Subjects’ Dominant Eye	Mean	Standard Deviation
OD:OS	38:19	
Spherical refraction (D)	0.28	0.41
Cylindrical refraction (D)	−0.40	0.24
VA_SC_ (logMAR)	−0.06	0.07
A.A. (D)	17.75	5.16

OD: right eye; OS: left eye; VA_SC_: visual acuity without correction; A.A.: amplitude of accommodation.

**Table 2:** Gender differences in accommodative response.

Viewing Distance	Mean ± SD		*p* Value *t* Test
	Male (*n* = 28)	Female (*n* = 29)	
0.4 m			
Accommodative response (D)	1.42 ± 0.46	1.28 ± 0.41	0.989
2.285 m			
Accommodative response (D)	0.46 ± 0.28	0.28 ± 0.29	0.064
DMS			
Accommodative response (D)	0.37 ± 0.20	0.38 ± 0.39	0.327

**Table 3:** Grade differences in accommodative responses.

Viewing Distance	Mean ± SD			*p* Value (AONVA)
	Lower Grade (*n* = 20)	Middle Grade (*n* = 25)	Higher Grade (*n* = 12)	
0.4 m				
Accommodative response (D)	1.31 ± 0.48	1.37 ± 0.38	1.35 ± 0.54	0.441
2.285 m				
Accommodative response (D)	0.33 ± 0.30	0.36 ± 0.30	0.47 ± 0.33	0.653
DMS				
Accommodative response (D)	0.30 ± 0.32	0.42 ± 0.33	0.45 ± 0.19	0.915

## Data Availability

The datasets used during the current study are available from the corresponding author.
